# Sacroiliac Joint Tuberculosis With Concomitant Iliopsoas Abscess Suggesting a Diagnostic and Therapeutic Challenge: A Case Report

**DOI:** 10.7759/cureus.73674

**Published:** 2024-11-14

**Authors:** Elissavet Symeonidou, Ariadni Fouza, Dimitrios Molyvas, Athina Pyrpasopoulou, Stergios Arapoglou, Maria S SidiropouIou, Chrysoula Gouta, Kalliopi Gianna, Sofia Deligiorgi, Alexandra Marneri, Chrysoula Nakou, Eleni Massa, Eleni Mouloudi, Konstantinos Mpallas

**Affiliations:** 1 5th Department of Surgery, Ippokrateio General Hospital/Aristotle University of Thessaloniki, Thessaloniki, GRC; 2 2nd Propaedeutic Department of Internal Medicine, Ippokrateio General Hospital of Thessaloniki, Thessaloniki, GRC; 3 Department of Radiology, Ippokrateio General Hospital of Thessaloniki, Thessaloniki, GRC; 4 Department of Pathology, Ippokrateio General Hospital of Thessaloniki, Thessaloniki, GRC; 5 Department of Adult Intensive Care Unit, Ippokrateio General Hospital of Thessaloniki, Thessaloniki, GRC

**Keywords:** antituberculosis treatment, extrapulmonary tuberculosis (eptb), psoas muscle abscess, sacroiliac joint tuberculosis, tuberculosis meningoencephalitis

## Abstract

Sacroiliac joint (SIJ) tuberculosis (TB) is an infrequent clinical entity, especially in developed countries. The symptoms are usually non-specific, and therefore it may mimic a variety of degenerative and non-degenerative diseases, hampering the diagnosis. An interesting case of SIJ infection with psoas abscess in a 77-year-old male is presented in the current article. The patient presented to the Infectious Diseases Clinic complaining of prolonged fever and difficulty walking. The fever was intermittent, it appeared usually at night, and it reached up to 39 degrees Celsius and was accompanied by chills. Magnetic resonance imaging (MRI) indicated SIJ osteomyelitis accompanied by two iliopsoas abscesses above the joint. Computed tomography (CT)-guided aspiration of the abscess was performed twice, but the microbiological culture did not grow any pathogens; Gene-X-Pert performed on the drained pus was negative. An open biopsy with drainage of the abscess cavities and bone biopsy was performed and set the diagnosis, and the anti-TB treatment was initiated. Shortly after the surgical procedure, the patient developed confusion and relapse of high-grade fever, and tubercular meningoencephalitis was diagnosed following an MRI of the brain, which revealed compatible lesions. The patient was intubated and transferred to the intensive care unit (ICU). A quadruple anti-TB regimen was administered. However, the patient’s condition deteriorated as he developed necrosis in the cortex and basal ganglia and the outcome was fatal. This article aims to raise awareness regarding this rare clinical entity, whose diagnostic and therapeutic management is particularly demanding.

## Introduction

Osteoarticular tuberculosis (TB) accounts for 1-5% of tubercular infections in general, usually affecting the spine and the joints of the lower limbs [1]. The sacroiliac joint (SIJ) is rarely affected by TB, with an incidence of up to 5-10% of all cases of skeletal TB [1]. The symptoms are usually nonspecific [2] and the differential diagnosis involves a variety of diseases, such as lumbar intervertebral disc herniation, ankylosing spondylitis, and myofascitis [3]. Thorough diagnostic evaluation including imaging with MRI and CT is essential to set the diagnosis of SIJ infection [3]. Serological tests for the diagnosis of latent TB infection are not adequate to commence anti-TB therapy in the absence of TB elsewhere, and further confirmation of the *Mycobacterium tuberculosis *with culture and histopathology of the affected area, specimens acquired either by CT-guided or open biopsy, is required [2-5]. Specifically, aerobic and anaerobic bacteria culture, fungal culture, acid-fast bacilli culture, polymerase chain reaction (PCR), drug susceptibility testing, and pathology should be performed on the material from the affected area in order to establish the diagnosis and initiate treatment [4,6]. Surgical management with excessive debridement, including abscess drainage and extraction of the affected bones, is required [7], followed by a long duration of anti-TB medication administration, usually 12 to 18 months [3,5]. TB meningoencephalitis appearing as a complication after an open biopsy has not been reported in the literature before.

## Case presentation

A 77-year-old Caucasian male presented to the Infectious Diseases Clinic complaining of prolonged fever and sciatica. The symptoms initiated two months ago. The fever could reach up to 39 degrees Celsius, appeared usually at night, and was accompanied by chills. It was intermittent and subsided by common antipyretics. He also mentioned difficulty walking and movement restriction of his right hip. Meanwhile, he visited three different doctors who treated him with different antibiotic regimes and non-steroid anti-inflammatory drugs, but both symptoms persisted.

Regarding his past medical history, he suffered from hypertension, hyperlipidemia, and prostate hyperplasia. He had undergone no previous surgeries in the past. He reported no allergies and had no previous admissions to the hospital for any reason. He was a non-smoker, otherwise fit man. No history of TB or contact with other patients with TB was mentioned. No travel history to any endemic area was reported. He was a plumber before he retired. He was admitted to the hospital for further diagnostic and therapeutic management. 

Upon admission, his vital signs were stable, with a blood pressure of 141/78 mmHg, 60 beats per minute, oxygen saturation rate of 98%, and temperature of 36.6 °C. Clinical examination revealed only right hip tenderness and movement restriction. White blood cells were 5300 K/mL (normal range 3800-10500 K/mL), with 74.6% neutrophils (normal range 45-75%), C-reactive protein (CRP) of 77.4 mg/dL (normal range <6), erythrocyte sedimentation rate (ESR) of 63 mm, and hemoglobin of 13.4 gr/dL. The plain radiograph of the chest was normal.

Thorough laboratory screening was performed to exclude possible viral or bacterial infection, as well as rheumatic diseases, as shown in Table 1. Blood cultures, including film array multiplex polymerase chain reaction (PCR) panel for a wide range of pathogens, were negative. His thyroid function was within normal limits, and all the serological cancer biomarkers were negative.

**Table 1:** Laboratory findings

	Laboratory findings	Normal range
White blood cells (K/mL)	5300	3800- 10500
Neutrophils	74.6%	45-75%
C-reactive protein (mg/dL)	77.4	<6
Erythrocyte sedimentation rate (mm)	63	0-15
Hemoglobin (gr/dL)	13.4	14-18
Brucella IgG (U/mL)	3.7	Negative <30
Ebstein-Barr IgM (AU/mL)	0.04	Negative <0.11
Ebstein-Barr IgG (AU/mL)	4.24	Positive > 0.21
CMV IgM IV	0.06	Negative < 0.7
CMV IgG (AU/mL)	69	Positive > 6
Toxoplasma IgM	Negative	
Toxoplasma IgG (IU/ml)	0	Negative <4
HBsAg (S/CO)	0.27	Negative <1
Anti-HBs (mIU/mL)	>823	Positive > 10, immunity > 25
Anti-HBc (S/CO)	12.78	Positive >1
Anti-HIV (human immunodeficiency virus)	Negative	
Widal test for typhoid and paratyphoid infection	Negative	
Rheumatoid factor RA/RF test U/mL	3.2	Negative <30
Antinuclear antibodies (ANA)	Negative	

**Figure 1:**
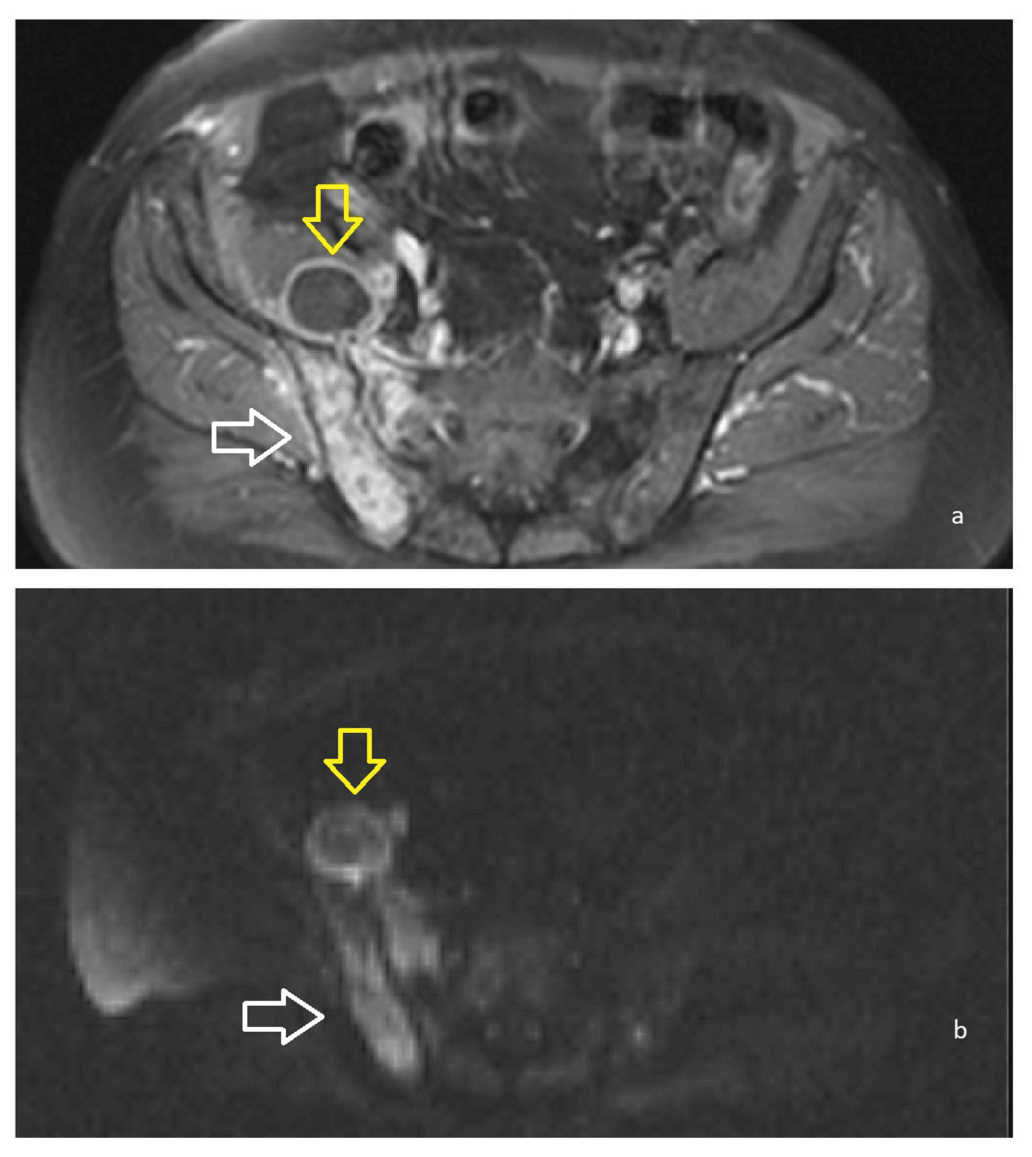
Magnetic resonance imaging (MRI) of the pelvis T1W1 sequence (a) and DWI (diffusion-weighted imaging, b): Depiction of abnormal increased enhancement and restricted diffusion of the right iliac bone, the right wing of the sacral bone, the right gluteus minimus and medius, and the ipsilateral piriformis muscle indicative of osteomyelitis (white arrow). A fluid-containing lesion with peripheral enhancement is imaged at the right iliopsoas muscle (yellow arrow).

An X-ray of the pelvis was performed, but it was nondiagnostic. Further diagnostic imaging was necessary. Magnetic resonance imaging (MRI) of the lumbar spine and the sacroiliac joints was performed before the admission, which indicated pathologic bone edema of the right sacroiliac joint, with two abscesses above the right SIJ and along the iliopsoas muscle, the largest one measuring 2.5 cm in size (Figure 1). Additional edema of the right iliopsoas muscle and the gluteus medius muscle were noticed. No signs of infection were reported regarding the lumbar spine. These findings confirm the diagnosis of septic SIJ arthritis.

**Figure 2:**
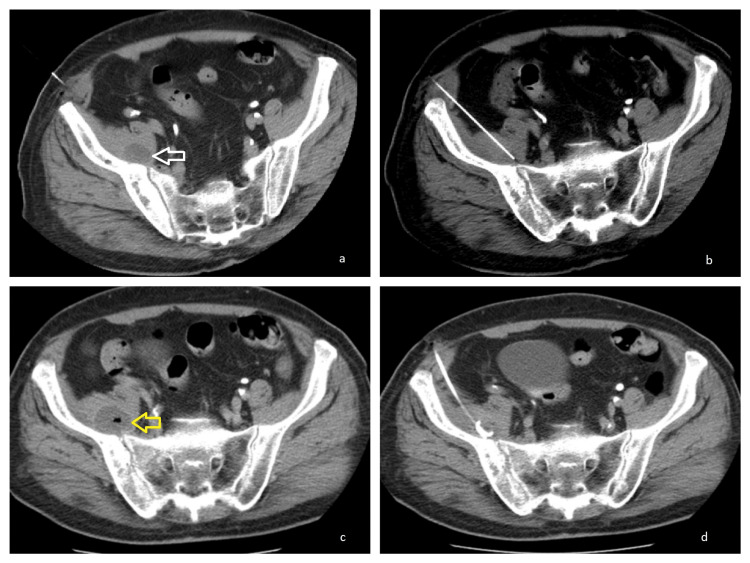
Computed tomography (CT) of the pelvis after intravenous contrast administration a and b: Guided paracentesis of the inflammatory fluid collection of the right iliac muscle (white arrow). c and d: Increase of the size of the inflammatory fluid collections of the right iliac muscle adjacent to the right iliac bone and the right sacral wing, currently measuring 3.6 x 2.5 x 6 cm, with increased density inside the cavity suggesting liquid rich in protein or hemorrhagic content, accompanied by peripheral enhancement (yellow arrow). Second CT-guided drainage of the iliac muscle fluid collection – anterior approach.

Amoxycillin/clavulanic 1 gr twice a day per os, followed by cefepime 400 mg once daily, was prescribed to the patient after the symptom onset and before his admission. Broad-spectrum empiric antimicrobial treatment with ciprofloxacin 400 mg twice daily IV and teicoplanin 400mg once a day IV was initiated upon his admission. This combination of antibiotics achieves activity against a wide range of pathogens, including gram-positive, gram-negative, and anaerobic bacteria. Computed tomography (CT) guided aspiration of the psoas abscess was performed (Figure 2a), and 15 ml of pus was aspirated. The aspirate was sent for bacterial and fungal cultures, but neither of them grew any pathogen. Neither acid-fast bacteria culture nor any molecular tests like Gene-X-pert were performed initially because there was no clinical suspicion. The patient remained febrile every day, with a temperature of up to 38.6 °C. For this reason, his empiric antibiotic treatment was escalated to meropenem 2 gr three times per day IV and daptomycin 400 mg once daily IV, and a second effort of CT-guided aspiration was performed, but the aspirate yielded no growth. In addition, the CT reveals deterioration and enlargement of both abscesses, which currently measured 3.6 x 2.5 x 6 cm and 3.15 x 1.55 x 2.4cm in size (Figure 2c), with increased density inside the cavity suggesting liquid rich in protein or hemorrhagic content, accompanied by peripheral enhancement after intravenous contrast administration. Dalbavancin 1.5 gr was also administered. Its long terminal half-life and its effectiveness for the treatment of osteomyelitis contributed to this decision, in an effort to enrich empiric antibiotic treatment.

**Figure 3:**
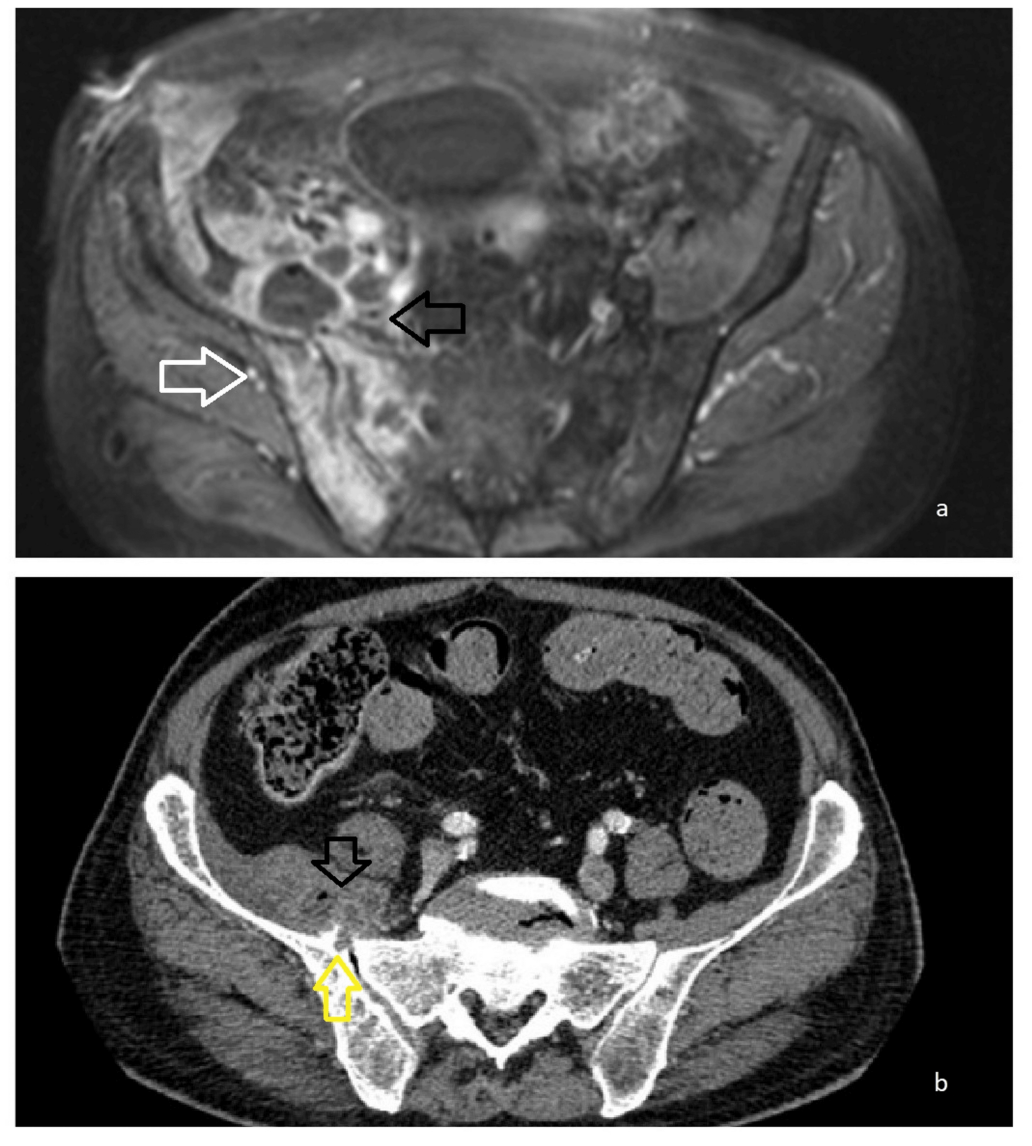
Imaging indicative of deterioration a. MRI of the pelvis T1WI FS +C. Increased and exacerbated enhancement of the right iliac bone and the right sacral wing indicative of osteomyelitis (white arrow). Multiple inflammatory fluid collections of the right iliopsoas muscle of increased size (black arrow). b. Pelvis CT post IV contrast administration. Smaller well-circumscribed inflammatory fluid collections posterior to the right iliopsoas muscle with air bubbles within (black arrow), in contact to the right iliac bone and right sacral wing. Erosion of the right iliac bone (yellow arrow).

A new MRI was performed during his admission, one month after the first one, which revealed edema of the right iliac bone, the right part of the sacrum, the right gluteus medius and gluteus minimus, and the right piriformis muscle, accompanied by diffusion restriction, findings consistent with the diagnosis of septic sacroiliitis (Figure 3a). Bone marrow edema represents a cellular infiltrate within the bone, and it is an early sign of sacroiliitis detected in MRI [4]. A new CT scan reveals erosion of the right iliac bone (Figure 3b). The abscess cavities upon the right iliopsoas muscle were described with peripheral enhancement and without diffusion restriction regarding their content.

A Quantiferon test was acquired and the result was positive, meaning a TB infection, but not necessarily active TB disease. Further microbiological and histopathological confirmation from the affected tissue was still necessary to begin anti-TB medication. Gene-X-Pert performed on the pus sample was negative. It was decided to proceed with an open biopsy, with an anterior extraperitoneal approach back to the iliopsoas muscle, during which the abscess cavity was opened and a 32Fr drainage catheter was placed. Both bacterial and fungal cultures of the pus were acquired and* Candida orthopsilosis *was detected, without any signs of bacilli. A second operation with the extraction of bone specimens was necessary. The retroperitoneal space was approached through a midline incision, after the mobilization of the cecum and the ascending colon. The iliac vessels and the right ureter were recognized and preserved. The abscess cavities upon the iliopsoas muscle were drained, and necrotic muscle fibers were extracted and sent to microbiology. The SIJ was further approached below the iliopsoas muscle and several bone specimens were obtained for microbiology and histopathology. Two drainage catheters were placed.

All the family members were tested for TB with the Quantiferon test, and all the results returned negative.

**Figure 4:**
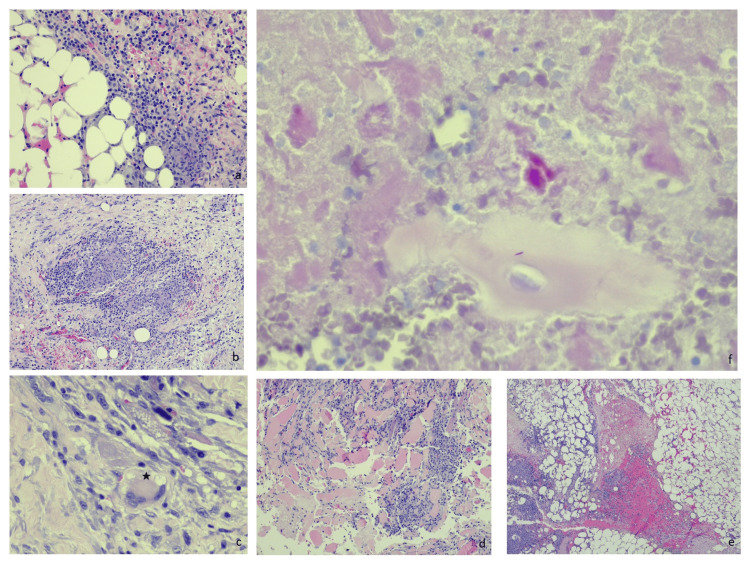
Histopathology report, hematoxylin-eosin staining a: 20x mixed inflammatory population consisting of lymphocytes, plasma cells, and neutrophils. b: 10x epithelioid granuloma consisting of epithelioid macrophages, lymphocytes, and fibrous tissue. c: 40x multinucleated giant cells Langhans-type. d. 10x bundles of striated muscle cells with dense inflammatory infiltration. e. 4x foci of caseous necrosis surrounded by epithelioid cells. f. 40x acid-fast microorganism detection on Ziehl-Neelsen stain.

On the fourth postoperative day (POD) after the second operation, the patient was intubated because of acute respiratory distress and altered state of consciousness. The new tissue and bone samples obtained from the right iliopsoas muscle and the right SIJ were tested by PCR for *M. tuberculosis* and were found to be weakly positive. Acid-fast bacteria culture was also positive for *M. tuberculosis*. The molecular antibiogram did not reveal resistance to any of the first-line antitubercular medications or fluoroquinolones. Histopathology from the necrotic tissue showed abundant infiltration of lymphocytes, plasmacytes, epithelioid histiocytes (Figures 4a and 4b), and several Langhans multinucleated giant cells (Figure 4c). The pathology report from the bone specimens described caseous necrosis in the center surrounded by granulomas (Figure 4e), while the Ziehl-Neelsen stain was positive for acid-fast bacilli (Figure 4f).

The patient commenced anti-TB treatment on POD 5 after his state deteriorated. The medication involved rifampicin/isoniazid 300 mg/150 mg 2 tb daily per os, rifampicin 300 mg once daily intravenous (IV), moxifloxacin 400 mg daily, and ethambutol 500 mg 3 tb daily per os, while the other empiric antibiotic regimes were stopped, and pyrazinamide was discontinued after the development of hepatotoxicity. Τhe quadruple regimen consisting of rifampicin, isoniazid, ethambutol, and pyrazinamide is the most common initial regimen for drug-susceptible TB, according to the World Health Organization (WHO). Moxifloxacin was administered additionally because of its effective bactericidal activity against* M. tuberculosis *and its high concentration in the cerebrospinal fluid.

**Figure 5:**
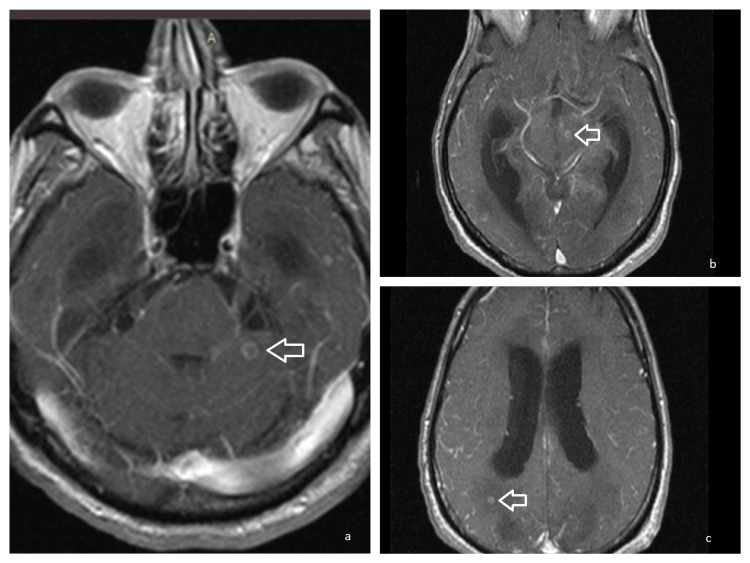
MRI of the brain T1WI Ring enhancing lesions (arrows) of the left anterior lobe of the cerebellum (a), the midbrain (b), and the right parietal lobe (c), typical for tuberculomas. T1WI: T1-weighted imaging

MRI of the brain was performed, which revealed areas of various sizes (less than 1 cm) and pathologic intensity in T2 and fluid-attenuated inversion recovery (FLAIR) imaging, as well as diffusion restriction in diffusion-weighted (DW) sequence, located in both the cerebellum, in the left occipital lobe, in the bilateral frontal and parietal lobes, in the pons, in basal ganglia, in the left hippocampus, in the midbrain, in the corpus callosum, and in the temporal poles, findings suggesting central nervous system (CNS) infiltration from the *M. tuberculosis* (Figure 5). After IV contrast administration, enhancement of the meninges was noticed, confirming the diagnosis of TB meningoencephalitis. CT of the thorax revealed aspiration pneumonia of the right lower lobe.

The patient remained in the intensive care unit (ICU). However, weaning from mechanical ventilation was impossible. MRI of the brain almost a month after the first one showed areas of cortical laminar necrosis in the left occipital lobe and laminar necrosis in the basal ganglia. His state was further deteriorated by hospital-acquired pneumonia, and the patient passed away after almost five months of hospitalization.

## Discussion

SIJ infection represents only 1-2% of septic arthritis in general [2]. The SIJ is a very uncommon site to be affected by TB, with an incidence of up to 5-10% of all cases of skeletal TB [1,2]. Its incidence is rising in undeveloped countries especially in Asia [3].

The synovial membrane that surrounds the joint makes it prone to TB infection, which happens usually via the hematogenous route, through the pelvic and paravertebral venous plexus of Batson [1]. Contiguous spread from the lumbar spine, along with the psoas muscle, suggests another possible route of infection [1].

In general, there are two types of psoas abscess. The primary one is caused by unrecognized staphylococcal bacteremia and it is more common in tropical areas. The secondary type results from intraabdominal or vertebral infections, such as diverticulitis, appendicitis, Crohn’s disease, and vertebral osteomyelitis [4]. The classic triad of symptoms involves fever, abdominal or back pain, and hip joint movement restriction, although it is present in less than 50% of the patients [4]. Sacroiliac joint TB presenting with secondary psoas abscess is an extremely rare surgical condition.

Pyogenic infection is the most common cause of sacroiliitis [1]. However, the differential diagnosis involves rheumatic, degenerative, and non-degenerative diseases such as lupus, ankylosing spondyloarthritis, inflammatory bowel disease, brucellosis, osteoarthritis [1], and neoplasmatic lesions [5]. Fungal infection and brucellosis should be also considered as possible causes of sacroiliitis [6]. TB remains a significant cause of subacute or chronic sacroiliitis in low- to middle-income countries, and its incidence is increasing due to human immunodeficiency virus infections, population migration, and immunosuppression [1]. Low socioeconomic status, intravenous drug use, and poor nutrition are predisposing factors, which are associated even with bilateral involvement [7]. Surprisingly, our patient was not immunosuppressed and he did not live in an endemic area.

Regarding clinical manifestation, SIJ TB might be asymptomatic, due to its deep location and limited range of motion, and as a result detected as an incidental finding. In advanced stages, it can cause severe pain and difficulty walking due to irritation of the nerve roots by the infection progress and iliopsoas muscle abscess formation [1]. The distribution of the pain might vary because of the proximity to the lumbosacral plexus and the location of the abscess [2]. Presacral abscesses may spread either to the inguinal and iliac fossa along the iliopsoas muscle, and then to the thigh and calf, or spread to the perineum through the perirectal space, to the ischial tuberosity along the sacrotuberous ligament, and even to the trochanter along the piriformis muscle, leading to the formation of sinus tracts [8]. The pain can be misinterpreted as lumbar instability, myofascitis, lumbar intervertebral disc herniation, and ankylosing spondylitis [3]. It may be accompanied by non-specific symptoms such as fatigue, weight loss, and anorexia, as well [1]. The vague character of the symptoms and the examination in the supine position may delay the diagnosis [2], which is usually set 16 to 19 months after the initiation of symptoms [3]. The low incidence of this disease in developed countries in combination with the lack of experience of the medical staff are also significant factors that hamper the diagnosis. Difficulty walking and abscess formation indicate severe destruction of the SIJ [3]. Instability of the SIJ and even dislocation of the pelvic ring are serious complications appearing as the disease develops if left untreated. In our patient, the diagnosis was established four months after the symptom onset, although two abscesses already existed.

The clinical examination might reveal tenderness along the affected joint, positive FABER (flexion, abduction, and external rotation) [4], and Gaenslen’s tests, whereas the straight leg raising test is usually negative [2]. Increased white blood cell count (WBC), C-reactive protein (CRP), and erythrocyte sedimentation rate (ESR) are common laboratory findings [9]. Our patient had elevated CRP and ESR.

MRI is more sensitive than CT in the early diagnosis of the disease, as it can reveal bone-marrow edema in the sacrum or ilium or both, soft tissue edema, joint space enlargement, abscesses, sinus tracts, destruction of iliac and sacral bones, capsular distention, and thin enhancing rim [1,2]. Ordinary X-rays are not sensitive for the diagnosis [10], especially in the early stages of the disease [6,11]. Radiological features of sacroiliitis such as erosion of the joints, loss of cortical margins, virtual joint space, and fusion of the joint, require months to develop and appear in conventional X-rays [4]. Loss of the cortical margins, widening of the joint space, erosions, and sclerosis are findings that appear later on plain radiographs [6]. Bone scans suggest a useful tool when diagnostic dilemmas exist [2]. However, no imaging can differentiate pyogenic from granulomatous SIJ infection [6]. In our case, X-ray was nondiagnostic, and MRI was extremely informative for diagnosing SIJ osteomyelitis, while CT was used additionally to assist with the biopsy and the drainage of the abscesses, as well as to exclude other pathologies.

A purified protein derivative (PPD) skin test is usually positive, but it is not specific to the current infection and it can be false negative on occasion [11]. Its specificity is low in areas with a high incidence of infection with nontuberculous mycobacteria or vaccination with BCG (Bacillus Calmette-Guérin) [5]. Identification of *M. tuberculosis* on Lowenstein-Jensen culture and a histopathological sample of the infected tissue are essential to set the diagnosis, especially when the TB is located only in the SIJ. CT-guided aspiration, a minimally invasive procedure performed under local anesthesia, is the proposed method to obtain a sample; however, an open biopsy might be inevitable when the aspiration result is negative. The paucibacillary nature of musculoskeletal TB makes acid-fast bacilli hard to identify [7]. PCR assay for TB in combination with susceptibility testing is necessary [4]. An obvious disadvantage of the CT-guided aspiration is the inability to provide histopathologic specimens. Ramlakan et al. [2] performed open biopsies on all patients. CT-guided aspiration was performed twice in our case. However, the cultures yielded no growth, and for this reason, an open biopsy was inevitable.

The anti-TB treatment is the cornerstone of therapeutic management, which leads to the relief of the symptoms and the prevention of possible complications. A quartile regimen consisting of isoniazid 4 mg/kg, rifampicin 10 mg/kg, pyrazinamide 15 mg/kg, and ethambutol 25 mg/kg should be initiated immediately after the diagnosis is established [7]. Multidrug resistance, which is very uncommon in these patients, might be a reason for treatment failure [4]. According to the drug susceptibility testing (DST), which was performed in our case, *M. tuberculosis* was sensitive to all the anti-TB medication. Regardless of that, the outcome was unfortunate.

Operative management seems unavoidable in cases of diagnostic uncertainty and the formation of an abscess [12], to prevent disease spread and fistula formation [1]. Specifically, Kim et al. [12] classified SIJ TB into four categories according to the severity of the infection. Types I and II are relatively mild and treated with anti-TB drugs only. For types III and IV with severe joint destruction, cystic degeneration of the ilium or sacrum, marginal sclerosis, and formation of abscesses, surgical treatment is required. Papangelopoulos et al. [5] suggested surgery in cases of abscess progression or persistence despite the anti-TB medication. Surgical therapy consists of drainage of the abscess, debridement of infected joint material, and arthrodesis with or without bone grafts, leading to increased blood flow of the surgical site and therefore decrease in the overall treatment period [1]. An anterior approach is a suitable option for abscesses located in the iliac fossa [1]. More artery injuries were noticed with the anterior approach [13]. Zhu et al. [3] proposed a combination of an anterior extraperitoneal approach for the blunt dissection of the abscess and the removal of pus and necrotic tissue, in combination with a posterior approach in the prone position, which involved the debridement of the infected bones and transplantation of a healthy bone graft to fill the gap and secure stability. An allogenic bone graft with or without internal fixation might be used instead [10]. Two weeks of anti-TB treatment administration, including rifampicin, isoniazid, and ethambutol, and a significant drop in ESR are recommended before the definitive operation, which involves excessive bone debridement, takes place [6,9].

The local irrigation of the affected areas with rifampicin and streptomycin powder was also suggested by the authors [3]. Tian et al. [10] applied carbolic acid, absolute ethanol, hydrogen peroxide, and normal saline locally to inactivate any lesions. The removal of pus and any necrotic tissue enables better penetration of the anti-TB medication [3]. The application of rifampicin-loaded calcium sulfate artificial bone grafts has shown some promising results, achieving shorter bone graft fusion time [14].

In cases of recovery, anti-TB treatment duration is between 12 and 18 months [3]. Patients with large abscess cavities including thin pus have poorer prognosis with higher recurrence rates [13]. During hospitalization, special attention should be given to the nutritional support of the patients and the possible need for blood transfusion [10]. Negative pressure wound therapy (NPWT), a non-invasive technique, has been proposed by Luo et al. [8] for the healing of SIJ TB sinus, as it decreases the mycobacteria load with the continuous suction of the pus, promoting the formation of granulation tissue at the same time.

Contamination of the CNS by *M. tuberculosis*, usually a result of hematogenous spread, may present as TB meningitis, which is the most common and the most fatal, tuberculoma, tuberculous abscess, and spinal TB [15]. This clinical manifestation of TB is associated with high mortality [15]. Regarding anti-TB medication, isoniazid, pyrazinamide, levofloxacin, and moxifloxacin achieve high concentrations in the cerebrospinal fluid, in contrast with rifampicin, streptomycin, and ethambutol, which require higher doses [15]. In our case, TB meningoencephalitis appeared as a complication following an open biopsy of the SIJ. Open biopsy was required in order to establish the SIJ TB diagnosis, which was set with acid-fast bacterial culture, molecular testing, and pathology. Before this confirmation, no anti-TB medication was administered, which might have led to the hematogenous spread of* M. tuberculosis* to the CNS.

## Conclusions

Early identification of SIJ TB is demanding, as nonspecific symptoms and lack of awareness from the medical community may postpone the diagnosis, resulting in long-term consequences, like the destruction of the SIJ. This becomes even more challenging when the patient is immunocompetent and does not live in an endemic area. Meticulous clinical examination and a thorough medical history are necessary. MRI is the most sensitive imaging method. Microbiological and histopathological confirmation of *M. tuberculosis* from the affected tissue is essential to begin the anti-TB medication in cases where TB infection is absent elsewhere (pulmonary and skeletal). The primary source of infection was not identified in the presented case. Prompt anti-TB medication and operative treatment including drainage and excessive debridement of the affected tissues are appropriate to increase the chances of survival and full recovery.
